# Improving Pain Literacy Among Future Educators: Contemporary Pain Neuroscience Education in Teacher Preparation

**DOI:** 10.1111/josh.70212

**Published:** 2026-07-31

**Authors:** Sue E. Curfman, William J. Best, Diana A. Harrison, Gary P. Austin, Darcie C. Finch

**Affiliations:** ^1^ Belmont University Nashville Tennessee USA; ^2^ Drexel University Philadelphia Pennsylvania USA

**Keywords:** adolescent health, chronic pain, pain literacy, pain neuroscience education, school health, teacher education

## Abstract

**Background:**

Persistent pain affects approximately one in five children and adolescents globally and is associated with school absenteeism, reduced academic engagement, psychosocial distress, and long‐term health consequences. Schools are important settings for developing health beliefs and self‐management behaviors, yet educators receive little formal preparation about pain.

**Contributions to Practice:**

This Practitioner's Perspective describes integration of a brief contemporary pain neuroscience education (CPNE) module into an undergraduate teacher preparation course. Two interactive sessions delivered by a physical therapist introduced pain as a protective nervous system output influenced by biological, psychological, and social factors. Participants demonstrated improved pain knowledge and beliefs, increased confidence discussing pain‐related concepts, and greater appreciation for the relevance of pain literacy to educational practice.

**Implications for School Health Policy, Practice, and Equity:**

Pain literacy may improve educator responses to student pain, reduce stigma, support appropriate participation, and promote equitable school environments. Physical therapists may be well positioned to collaborate with teachers, school nurses, counselors, coaches, and other school‐based professionals.

**Conclusions:**

Teacher preparation programs may represent an upstream opportunity to improve pain literacy, support student health and learning, and contribute to the prevention of persistent pain.

Persistent pain in children and adolescents is increasingly recognized as a major public health concern with profound implications for educational systems. A recent systematic review and meta‐analysis of 119 population‐based studies across 70 countries estimated the overall prevalence of persistent pain in children and adolescents to be 20.8%, or approximately 1 in 5 youth [1]. Pediatric persistent pain is situated within a broader global context in which the burden of persistent pain is increasing [2]. Together, these findings underscore the ongoing and inadequately addressed public health significance of pediatric persistent pain.

Pain medication use among young people is a significant public health concern. Similar to trends observed in adults, opioid prescribing to children and adolescents increased during the early years of the opioid epidemic before beginning a decline in recent years [3, 4]. Despite these reductions, opioid exposure during adolescence remains concerning. Prescription opioid use has been associated with an increased risk of future misuse and nonmedical opioid use in early adulthood [5, 6]. Legitimate opioid use before high school graduation is independently associated with a 33% increase in the risk of future opioid misuse after high school [6]. Concern about opioid use is heightened by the continued rise in overdose deaths driven largely by synthetic opioids such as fentanyl in the United States [7, 8].

At the same time, the use of over‐the‐counter analgesics among children and adolescents has increased, suggesting that the treatment of pain may be shifting rather than resolving, normalizing frequent use of medications for pain [9]. Although over‐the‐counter medications such as acetaminophen and nonsteroidal anti‐inflammatory drugs are often perceived as safer alternatives to prescription opioids, they are not without risk. Adverse effects are well documented yet frequently underrecognized in discussions of the treatment of pediatric pain [10].

Beyond concerns related to medical management, there are consequences of persistent pain during childhood that impact children's ability to engage in education. Children and adolescents with persistent pain experience higher rates of school absenteeism, reduced academic engagement, impaired social development, sleep disruption, anxiety, and depression [11, 12, 13]. Longitudinal studies demonstrate that pediatric persistent pain frequently persists into adulthood, contributing to ongoing healthcare utilization, diminished vocational attainment, and increased reliance on public assistance [14, 15].

Together, these factors highlight a broader issue of inadequate pain literacy among children and the adults who influence their health behaviors. Pain literacy broadly refers to an individual's capacity to understand pain, evaluate pain‐related information, and make informed decisions regarding the treatment of pain [16, 17, 18]. Limited pain literacy may be associated with maladaptive beliefs about pain, increased fear of movement, greater reliance on passive or pharmacological approaches, delayed use of evidence‐based self‐management strategies, and stigma toward individuals living with persistent pain [16, 18, 19, 20]. Maladaptive pain beliefs among adults may also influence how children's pain is interpreted and managed [21]. While pain literacy initiatives have largely focused on healthcare settings, far less attention has been given to educational systems, although schools are among the primary environments in which children develop health beliefs, coping behaviors, and self‐management skills [22, 23]. Emerging school‐based pain education initiatives delivered by physical therapists suggest that improving pain knowledge during childhood and adolescence may positively influence pain beliefs, health behaviors, and medication use [24, 25]. Improving pain knowledge among educators may therefore represent an important and underexplored opportunity to influence how pain is understood and managed during childhood and adolescence.

Students experiencing pain may encounter skepticism, excessive alarm, or misinterpretation of symptoms, responses that can inadvertently reinforce fear‐based beliefs and exacerbate functional limitations [11, 26]. This is relevant not only for students living with persistent pain but also for the many acute pain experiences that occur in schools, including sports injuries, falls, headaches, musculoskeletal injuries, and everyday bumps, cuts, and bruises. Teachers are often among the first adults to respond to these events, and their reactions may shape how children experience pain, engage in activity, and recover. Early responses that promote reassurance, appropriate support, and functional recovery may help reduce maladaptive pain beliefs that contribute to the transition from acute to persistent pain.

Contemporary pain neuroscience education (CPNE) represents a paradigm shift from traditional biomedical models of pain. CPNE conceptualizes pain as a protective output of the nervous system influenced by biological, psychological, and social factors. Extensive research demonstrates that CPNE improves pain knowledge, reduces maladaptive beliefs, and supports functional recovery across populations [27, 28, 29]. Emerging evidence from school‐based interventions by physical therapists suggests that CPNE delivered during childhood and adolescence can positively influence pain‐related behaviors, school attendance, and healthcare utilization [24, 25].

This Practitioner's Perspective explores teacher preparation programs as an upstream opportunity to improve pain knowledge among future educators. By equipping future teachers with an accurate, evidence‐based understanding of pain, educational systems may contribute not only to broader public health efforts aimed at mitigating persistent pain and opioid risk, but also to more effective support for students who are already experiencing persistent pain. Teachers who understand pain through a contemporary biopsychosocial lens may be better prepared to respond with empathy, reduce stigma, support appropriate pain treatment strategies within the school context, and help create learning environments in which students in pain feel understood, accommodated, and able to participate more fully.

## 1 Presentation of the Practice

The practice described here occurred within an undergraduate child development and education course at a university in the southeastern United States. The course enrolled first‐year undergraduate students majoring in education, many of whom were preparing for careers in elementary and secondary education. A total of 15 students completed both the pre‐ and post‐module assessments and were included in the analysis. Recognizing the intersection between health, learning, and classroom participation, a brief CPNE module was integrated into the existing curriculum.

The goal of the CPNE module was not to train teachers as healthcare providers, but rather to provide future educators with foundational knowledge about pain that could inform responses to both acute and persistent pain experiences, classroom interactions, and collaboration with families and school health professionals. Because teachers frequently respond to everyday injuries and pain reports in school settings, the module emphasized how adult reactions to pain can either reinforce fear and avoidance or promote reassurance, resilience, and appropriate recovery.

The CPNE module consisted of two interactive 75‐min sessions delivered by a physical therapist clinician and educator with more than 15 years of clinical experience with patients with persistent pain and teaching contemporary pain neuroscience. Instructional strategies included interactive lecture, short educational videos, guided discussion, real‐world examples, and reflective exercises. Core topics included distinctions between acute and persistent pain; the role of the nervous system in pain perception; the influence of emotions, stress, social factors, and context on pain; and the importance of movement and reassurance in recovery [28, 29]. The module also included an overview of general pharmacologic and non‐pharmacologic pain treatment approaches. Students were introduced to the magnitude of the opioid crisis, risks associated with opioid exposure, increasing use of over‐the‐counter analgesics among youth, and the role of movement, reassurance, stress management, sleep, and gradual return to activity as evidence‐informed non‐pharmacological strategies. The purpose was not to train teachers to provide medical advice, but to broaden their understanding of contemporary pain interventions.

Throughout the sessions, students were encouraged to reflect on their own experiences with pain and to consider how pain‐related beliefs might influence their future interactions with students. This reflective component was critical in fostering empathy and reducing stigma. By normalizing pain as a common human experience and emphasizing variability in pain responses, the module aimed to shift students' conceptualizations away from judgment and toward understanding.

From a practice perspective, the module was feasible to implement within existing coursework and required minimal additional resources. Student engagement was high, and informal feedback suggested that the content was perceived as relevant, meaningful, and applicable to future teaching roles.

### 1.1 Practice Outcomes

#### 1.1.1 Changes in Pain Knowledge and Beliefs

Data were available for all 15 participants across both the Concept of Pain Inventory for Adults (COPI‐Adult) and revised Neurophysiologic Pain Questionnaire (rNPQ) measures. Following participation in the two‐session CPNE module, undergraduate education majors demonstrated meaningful improvements in their understanding of pain and in their beliefs about how pain is experienced and managed. These changes were observed across both global measures of pain beliefs as assessed by the COPI‐Adult and pain knowledge assessed by the rNPQ, suggesting that the module influenced not only factual understanding but also broader conceptual frameworks relevant to educational practice.

**FIGURE 1:**
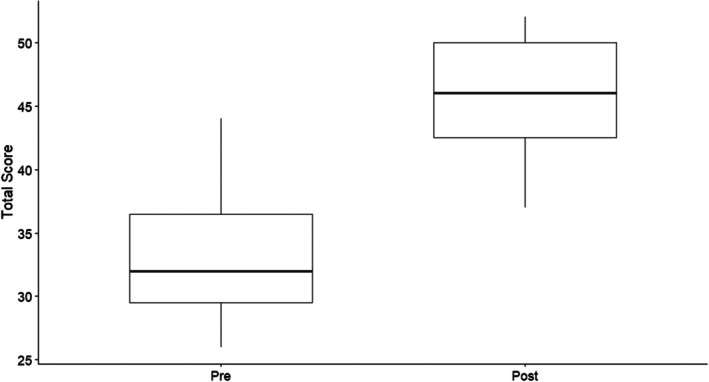
COPI scores pre versus post (*N* = 15).

Scores on the COPI‐Adult increased from before to after the module, with improvements representing a large effect size. Every participant demonstrated improvement following the sessions (Figure 1), indicating a consistent shift toward more accurate, biopsychosocial understandings of pain. Improvements remained robust regardless of participants' baseline beliefs. In other words, students with more limited or more accurate pain beliefs at the outset benefited similarly from the module, suggesting broad applicability within teacher preparation contexts.

**FIGURE 2:**
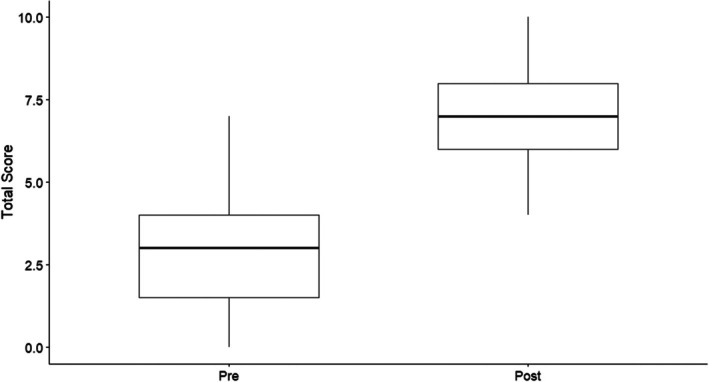
rNPQ scores pre versus post (*N* = 15).

Knowledge of pain neuroscience, as measured by scores on the rNPQ, also improved following the CPNE sessions (Figure 2). Most participants demonstrated increased scores after the module, with the largest gains observed among those who began with the least prior knowledge. This pattern suggests that CPNE may be particularly effective for individuals with limited exposure to pain science—an important consideration given that most preservice teachers receive little formal education related to pain. While two participants showed no change in rNPQ scores, no participant demonstrated a decline, reinforcing the overall positive and low‐risk nature of the educational intervention.

Taken together, these findings suggest that even brief, classroom‐based CPNE can meaningfully improve both pain knowledge and pain‐related beliefs among future educators. Improvements in pain beliefs were robust regardless of baseline beliefs, while gains in pain knowledge were most pronounced among those with the least prior exposure to pain science.

#### 1.1.2 Patterns of Change Across Pain Concepts

Examination of individual COPI‐Adult items revealed that improvements were not confined to a single concept, but rather spanned all domains assessed by the instrument (Table 1). Median scores increased on every item following the module, with large effects observed across questions. These items reflect key principles of contemporary pain science that are directly relevant to educational settings, including the role of emotions in pain, the influence of the brain and nervous system, and the importance of movement and activity in recovery.

**TABLE 1:** COPI‐adult item analysis (*N* = 15).

COPI questions	Pre median/IQR	Postmedian/IQR	*p*	Effect size
1. Feeling sad can make you feel more pain.	3 (±1.5)	4 (±1.0)	0.01*	0.85
2. Doing something you enjoy can make you feel less pain.	3 (±2.0)	4 (±1.0)	0.04*	0.56
3. Feeling pain for a long time can make the brain more sensitive to warning messages.	2 (±0.5)	4 (±2.0)	0.03*	0.56
4. You can feel a lot of pain even when an injury is small.	3 (±2.0)	4 (±1.0)	0.02*	0.66
5. Learning about pain can help you to feel less pain.	2 (±1.0)	4 (±1.0)	0.01*	0.75
6. You can have an injury and feel no pain.	3 (±2.0)	4 (±0.5)	0.02*	0.66
7. The brain can make pain better or worse.	3 (±2.0)	4 (±1.0)	0.03*	0.61
8. You can feel a little bit of pain even when an injury is big.	3 (±2.0)	4 (±1.0)	0.02*	0.65
9. Pain usually feels better if you move your body a little bit more each day.	2 (±0.5)	3 (±1.0)	0.01*	0.82
10. The brain processes lots of details before you feel pain.	2 (±0.5)	4 (±1.0)	0.01*	0.87
11. Resting for a long time can make pain worse.	2 (±0.0)	3 (±0.5)	0.01*	0.75
12. Pain is a feeling that is made by the brain.	2 (±1.0)	4 (±1.0)	0.03*	0.68
13. Pain can be too protective if it stops you getting moving again.	2 (±0.5)	4 (±1.0)	0.01*	0.85

* Denotes a significant (< 0.05) *p* value.

The direction of change was uniform across all questions. This consistency suggests that participants did not simply memorize isolated facts, but instead developed a more integrated understanding of pain as a complex, context‐sensitive experience. From a practice standpoint, this breadth of change is particularly important, as teachers encounter pain‐related behaviors in diverse forms and contexts within the classroom.

Analysis of individual rNPQ items further clarified which aspects of pain neuroscience were most influenced by the module (Table 2). Four items demonstrated marked improvements in the proportion of correct responses following the sessions. These items corresponded to foundational concepts, including the role of the nervous system in processing danger signals and the brain's role in determining when pain is experienced. These concepts are often counterintuitive to individuals without healthcare training and are frequently misunderstood in educational and community settings. Improvement in these areas suggests that the module successfully addressed common misconceptions that can shape how adults respond to students in pain.

**TABLE 2:** RNPQ item analysis (*N* = 15).

rNPQ questions	Pre % correct	Post % correct	*p*
1. It is possible to have pain and not know about it.	0%	6.67%	1
2. When part of your body is injured, special pain receptors convey the pain message to your brain.	0%	0%	NA
3. Pain only occurs when you are injured or at risk of being injured.	60%	80%	0.38
4. When you are injured, special receptors convey the danger message to your spinal cord.	20%	80%	0.01*
5. Special nerves in your spinal cord convey ‘danger’ message to your brain.	33.33%	86.67%	0.01*
6. Nerves adapt by increasing their resting level of excitement.	26.67%	66.67%	0.07
7. Chronic pain means that an injury hasn't healed properly.	33.33%	66.67%	0.13
8. The body tells the brain when it is in pain.	6.67%	33.33%	0.22
9. Nerves adapt by making ion channels stay open longer.	0%	40%	0.03*
10. Descending neurons are always inhibitory.	0%	13.33%	0.50
11. Pain occurs whenever you are injured.	33.33%	66.67%	0.18
12. When you injure yourself, the environment that you are in will not affect the amount of pain you experience, as long as the injury is exactly the same.	46.67%	80%	0.18
13. The brain decides when you will experience pain.	26.67%	86.67%	0.01*

* Denotes a significant (< 0.05) *p* value.

#### 1.1.3 Interest, Perceived Usefulness, and Confidence to Teach

Participants entered the module already expressing moderate interest in learning about pain and perceiving the topic as potentially useful for their future careers as educators. As a result, overall median ratings for interest and perceived usefulness did not change significantly following the module. However, closer examination of response patterns revealed meaningful shifts that are relevant from a practice perspective.

Prior to the CPNE sessions, very few participants reported high levels of interest in learning about pain. After the module, nearly half of the participants rated their interest at higher levels, suggesting that exposure to CPNE may broaden engagement even among those who initially viewed pain as outside the scope of teaching. Similarly, while median ratings of perceived usefulness remained stable, nearly half of the participants reported an increased sense of how pain‐related knowledge could be applied in their future professional roles. These findings suggest that CPNE may refine and deepen perceptions of relevance, even when baseline attitudes are already positive.

The most notable change occurred in participants' confidence to teach pain‐related concepts. Following the module, participants reported significantly greater confidence in their ability to discuss pain with future students, particularly when additional training or resources were available. Whereas only a small proportion initially felt confident in teaching about pain, the majority indicated high confidence when considering the possibility of further preparation. This finding is particularly relevant for school health practice, as it highlights both the promise of CPNE and the importance of ongoing professional development rather than one‐time exposure.

#### 1.1.4 Participant Perspectives and Lived Experience

Qualitative responses provided important context for the observed quantitative changes and reinforced the relevance of CPNE for future educators. Two open‐ended survey questions asked participants to: (1) describe what impacted them most during the pain module and (2) explain how they anticipated applying what they learned about pain in the future. When reflecting on what impacted them most, participants frequently described gaining a new appreciation for the complexity and interconnectedness of pain. Statements such as “Pain is intertwined with so much in our lives” and “Pain is so much more than just an injury” reflected a shift away from simplistic, injury‐focused explanations toward a more holistic understanding.

Many participants found learning about the roles of mindset, perception, and context to be especially impactful. This understanding resonated on both personal and professional levels, as students reflected on their own experiences with pain while also considering how these ideas apply to children in school settings.

Several participants explicitly described integrating newly learned strategies into a broader “toolbox” for understanding and responding to pain. Others highlighted how reframing their own pain experiences made those experiences feel more manageable. These reflections underscore the potential for CPNE to influence not only knowledge but also empathy, self‐awareness, and future teaching practices.

#### 1.1.5 Summary of Practice‐Relevant Findings

Overall, the CPNE module was associated with consistent improvements in pain knowledge, beliefs, and confidence among undergraduate education majors. The combination of quantitative and qualitative findings suggests that brief, structured pain education can meaningfully shift how future teachers conceptualize pain and their role in responding to it.

## 2 Discussion

This *Practitioner's Perspective* highlights the promise of teacher preparation as an upstream intervention point for pain education. Teachers often serve as first responders to students' expressions of pain, whether through requests to leave class, reduced participation, behavioral changes, or common acute pain experiences such as headaches, sports injuries, musculoskeletal complaints, and everyday bumps, cuts, and bruises. When teachers lack understanding of pain, these experiences may be misinterpreted, potentially leading to disciplinary responses, excessive fear‐based messaging, or unnecessary exclusion from learning activities [26]. In contrast, teachers equipped with foundational pain knowledge may be better prepared to respond with reassurance, encourage appropriate activity, and support healthier recovery behaviors.

Teachers equipped with a biopsychosocial understanding of pain may be better able to engage learners by promoting safety, fostering empathy, validating student lived experiences, and encouraging adaptive coping strategies. CPNE does not require teachers to diagnose or treat pain; rather, it provides a framework for understanding why pain exists and, in some cases, persists, and how interactions informed by CPNE can promote recovery [28, 29]. This shared conceptual framework may also enhance collaboration and coherence between teachers, school nurses, counselors, families, coaches, and other healthcare professionals working within school systems.

CPNE may also extend beyond informal teacher‐student interactions. Although contemporary pain education has historically been underemphasized in the preparation of many health care students and professionals, numerous health professions are increasingly recognizing contemporary pain neuroscience as integral components of training [30, 31, 32, 33, 34]. This broader shift suggests that schools may also have an opportunity to support pain literacy through developmentally appropriate, interdisciplinary approaches. We envision educator involvement occurring along a continuum that includes both individualized responses to student pain and more structured educational opportunities. For example, pain education concepts could be incorporated into existing health, science, or physical education curricula where topics related to the nervous system, health behaviors, and self‐management are already discussed. Contemporary pain neuroscience concepts may also be reinforced by school nurses, counselors, coaches, and other school‐based health professionals. Importantly, we are sensitive to the reality that many teachers already feel overextended, and we do not suggest that pain education should become an additional burden without thoughtful integration into existing responsibilities and curricula. Rather, these findings highlight multiple potential pathways for implementation.

The integration of CPNE into teacher preparation aligns with broader educational initiatives emphasizing social–emotional learning, trauma‐informed pedagogy, and whole‐child development. Pain frequently co‐occurs with stress, trauma exposure, and mental health challenges, particularly among students from marginalized communities [35, 36]. Teachers who understand these intersections may be better positioned to respond in ways that reduce harm and promote equity.

Importantly, positioning teachers as partners in pain education does not absolve healthcare systems of responsibility. Instead, it reflects a public health approach that recognizes the value of prevention, early intervention, and community‐based education. By introducing accurate pain concepts early in life, schools may help shape lifelong beliefs and behaviors that encourage evidence‐informed understanding of pain beyond medication‐focused responses and potentially reduce risk factors associated with persistent pain development.

## 3 Implications for School Health Research

Further research is needed to evaluate the effectiveness and sustainability of CPNE delivered through teacher preparation and professional development programs. As an initial pilot study involving a small sample of preservice teachers, these findings should be interpreted cautiously and expanded through larger studies involving more diverse samples of preservice teachers as well as in‐service teachers currently working in school systems. Future studies should examine long‐term outcomes related to teacher beliefs, classroom practices, student engagement, school attendance, and responses to both acute and persistent pain experiences. Research designs incorporating comparison groups and longitudinal follow‐up will be essential.

Additional research should explore implementation across diverse educational contexts, including urban and rural schools, under‐resourced districts, and culturally diverse populations. Future work should also examine whether preservice teachers and in‐service teachers can effectively deliver pain‐related content, how teacher‐delivered CPNS education compares with instruction delivered by healthcare professionals such as physical therapists, and which implementation models are most feasible, effective, and sustainable in school settings. Understanding how CPNE can be adapted to meet the needs of different communities will be critical for promoting equity and scalability.

Future studies examining pain neuroscience education as a strategy to improve pain literacy should evaluate outcomes that extend beyond improvements in educator knowledge and beliefs. Although measures such as the COPI and rNPQ provide important insight into conceptual understanding, they do not capture whether increased pain literacy translates into meaningful behavioral, educational, and health‐related outcomes. Future research should examine students' own understanding of pain and their perceptions of how pain influences daily functioning. School‐related outcomes are particularly important, given evidence that persistent pain is associated with impaired school functioning, including absenteeism, difficulty concentrating, reduced classroom participation, lower academic engagement, and social challenges [11, 13]. Additional educational outcomes may include academic performance, participation in extracurricular activities, and teachers' perceptions of how improved pain education influences classroom interactions, student engagement, and learning. Healthcare‐related outcomes should also be considered, including healthcare utilization, school nurse visits, and use of prescription and over‐the‐counter pain medications. Because one potential goal of early pain education is to promote healthier responses to acute pain, future longitudinal studies should examine whether pain education interventions influence the transition from acute to persistent pain. Mental health outcomes—including anxiety, depression, stress, resilience, and coping—may also be relevant given the well‐established relationship between pain and psychological well‐being. Finally, because teaching is an emotionally demanding profession with high rates of stress and burnout, future studies should explore whether improved pain literacy influences teachers' own pain experiences, stress levels, burnout, job satisfaction, and sense of personal agency in managing pain. Collectively, these broader outcome measures may provide a more comprehensive understanding of the educational, health, and public health impact of school‐based pain literacy initiatives.

## 4 Implications for School Health Policy, Practice, and Equity

From a policy perspective, integrating foundational pain education into teacher preparation programs warrants consideration as part of broader efforts focused on whole‐child development, mental health promotion, and prevention‐oriented health education. Schools increasingly serve as important settings for health promotion, particularly for students who face barriers to healthcare access and health literacy development [17, 22]. Health literacy developed during childhood has been linked to improved health decision‐making and long‐term health behaviors, suggesting that schools may be an important setting for early pain literacy development [17, 22]. However, this pilot study should not be interpreted as sufficient evidence to support widespread curricular mandates. Rather, it provides preliminary evidence that future educators are capable and interested in learning CPNE. This is particularly important because many educators may also navigate their own pain experiences, which can influence how they respond to students experiencing pain. Future research should determine whether pain education should be more broadly integrated into teacher preparation curricula and identify the most effective dosage and format for implementation.

Core components of educator‐focused pain education should remain practical, developmentally appropriate, and aligned with the scope of educational practice. Potential topics may include understanding acute versus persistent pain, pain as a construct of the brain, the biopsychosocial contributors to pain, the influence of stress and emotions on pain experiences, common misconceptions about pain, strategies for reducing fear‐based messaging, and approaches that encourage appropriate movement, participation, and supportive communication. These concepts align closely with established CPNE frameworks shown to improve pain knowledge, reduce maladaptive beliefs, and improve functional outcomes [27, 28, 29]. Importantly, educators should not be expected to diagnose or treat pain, but rather to recognize pain‐related challenges and respond in ways that support students while facilitating appropriate referral when needed.

Physical therapists may be particularly well‐positioned to contribute to this educational effort, given their training in the biopsychosocial nature of pain and the frequent focus on pain in clinical practice [30, 31, 32, 33, 37, 38, 39]. Prior research demonstrates that physical therapists have played successful roles in delivering CPNE in both clinical and community settings [24, 25, 28, 40, 41]. At the same time, school nurses, counselors, psychologists, athletic trainers, occupational therapists, and other school‐based health professionals may also play important roles in reinforcing pain literacy within school systems.

In practice, pain education may be implemented through multiple pathways. At an informal level, teachers may apply pain literacy principles during everyday interactions with students experiencing headaches, sports injuries, musculoskeletal complaints, or other common pain experiences. At a broader population level, pain concepts could also be integrated into existing health, science, or physical education curricula. This flexible approach may be particularly important given concerns about increasing demands placed on educators.

## 5 Conclusions

Educating future teachers about contemporary pain neuroscience represents a feasible and prevention‐oriented strategy to support student health and learning. This Practitioner's Perspective suggests that CPNE can be meaningfully integrated into teacher preparation programs, improving pain knowledge and beliefs while increasing interest and confidence among future educators to convey this information to students. Teachers may apply this knowledge through both individualized interactions with students experiencing pain and, potentially, through broader school‐based educational initiatives. By positioning educators as informed partners in pain literacy, schools may contribute to improved responses to both acute and persistent pain, reduced stigma, healthier recovery behaviors, and broader public health benefits related to persistent pain prevention.

## Funding

The authors have nothing to report.

## Ethics Statement

The authors have nothing to report.

## Consent

Informed consent was obtained from all participants prior to the study.

## Conflicts of Interest

The authors declare no conflicts of interest.

## Data Availability

The data that support the findings of this study are openly available in Mendeley Data at http://doi.org/10.17632/k7jpghm98c.1 [42].
